# The role of fibrocytes in fibrotic diseases of the lungs and heart

**DOI:** 10.1186/1755-1536-4-2

**Published:** 2011-01-10

**Authors:** Ellen C Keeley, Borna Mehrad, Robert M Strieter

**Affiliations:** 1 Division of Cardiology, Department of Medicine, University of Virginia, Lee Street, Charlottesville, Virginia 22908, USA; 2 Pulmonary and Critical Care Medicine, Department of Medicine, University of Virginia, Lee Street, Charlottesville, Virginia 22908, USA

## Abstract

Fibrosis is the end result of a complex series of events that follow tissue injury and inflammation. Pathophysiologic fibrosis results in permanent scar formation, and can impair organ function. Fibrocytes are circulating, bone-marrow-derived progenitor cells that traffic from the bone marrow to the injured organ via the bloodstream, where they differentiate into fibroblasts and myofibroblasts, and play a pivotal role in both physiologic and aberrant fibrosis. In this review, we focus on the contribution of fibrocytes to fibrotic diseases of the lungs and the heart, including interstitial lung diseases, asthma, pulmonary hypertension, atherosclerosis and ischemic cardiomyopathy.

## Introduction

Fibrosis is the end result of a complex series of events that follow tissue injury and inflammation. If this process is faulty, excessive matrix deposition occurs and normal tissue is replaced with permanent scar, resulting in organ dysfunction. Fibrocytes are circulating bone-marrow-derived progenitor cells that were first identified in the context of an experimental model of wound repair [1], and have since been shown to play a pivotal role in both normal and aberrant healing in humans [2]. The striking similarities seen in fibroproliferative processes involving both the lungs and the heart has brought about the possibility of a common etiologic mechanism [3]. In this review, we focus on a unique mesenchymal progenitor cell, the fibrocyte, as the potential common denominator in fibrotic diseases affecting the lungs and the heart.

### The fibrocyte

Fibrocytes were originally described when investigators noted the early appearance of spindle-shaped cells that expressed collagen, procollagen and CD34 in an experimental skin wound repair model [1]. Their appearance in the wound chamber, within 1 day after injury, was much earlier than would be expected by entry of fibroblasts from the surrounding skin, and therefore these cells were thought to originate from the circulation. Thus, the investigators coined the word 'fibrocyte', a combination of 'fibroblast' and 'leukocyte'. In healthy humans, fibrocytes comprise 0.1-1.0% of nucleated cells in peripheral blood, and have been found in a variety of tissues in both healthy and diseased states [4-6].

Fibrocytes perform diverse functions that are important in normal tissue repair and fibrosis. In addition to mediating fibrogenesis by secreting inflammatory cytokines, chemokines and growth factors [4,7], fibrocytes also constitutively produce extracellular matrix proteins (collagen I, collagen III and vimentin) and secrete matrix metalloproteinases [7]. In addition, they play a role in the recruitment and activation of T cells as antigen-presenting cells [8], serve as the contractile force of wound closure via α-smooth muscle actin (SMA) expression [4,9], and promote angiogenesis [10].

### Bone marrow origin, differentiation and homing of fibrocytes

#### Bone marrow origin

Current data support the hypothesis that fibrocytes are derived from the bone marrow. Fibrocytes express the hematopoietic stem cell antigen CD34, the common leukocyte marker CD45, the major histocompatibility complex II, the myeloid markers CD11b and CD13, and several fibroblast markers including vimentin, collagen I, collagen III and fibronectin [1,5,6]. Fibrocytes also express several chemokine receptors and adhesion molecules [4,6]. Only a subset of circulating fibrocytes express CD34, and the expression of both CD34 and CD45 on fibrocytes decreases when the cells are cultured or after they enter tissue. Because there is no single marker unique for the identification of fibrocytes, the coexpression of collagen production and CD34 or CD45 has been commonly used.

However, the progressive loss of CD34 and the eventual loss of CD45 can lead to an underestimation of fibrocyte numbers. To address this issue, investigators studied a comprehensive panel of markers and discovered that human fibrocytes, but not peripheral blood mononuclear cells, tissue macrophages or fibroblasts, express the unique combination of CD45RO, 25F9 and S100A8/A9 but not PM-2K [11]. Their findings suggest that discrimination between these cells is possible, and may facilitate studying the role of fibrocytes in fibrotic diseases [11].

#### Differentiation

In culture, fibrocytes express CD34, CD45, collagen I and vimentin. After exposure to transforming growth factor (TGF)-β or endothelin-1, fibrocytes differentiate into myofibroblast-type cells with expression of α-SMA, production of fibronectin and collagen, and loss of expression of CD34 and CD45 [4-6,9,12]. Other factors shown to either promote or inhibit fibrocyte differentiation include serum amyloid P [13,14], cytokines [9,15-17], peroxisome proliferator-activated receptor-γ agonists [15]; leukotrienes [18], immunoglobulin G [19], and gadolinium-containing contrast agents [20].

The differentiation of fibrocytes into myofibroblasts has been studied in a variety of models including a wound repair model [21] and a lung fibrosis model [22]. These authors reported that bone-marrow transplantation from green fluorescent protein (GFP) transgenic animals to wild-type animals resulted in the presence of cells in the target organ that coexpressed GFP and α-SMA, suggesting that the myofibroblasts were derived from bone marrow [21,22]. In addition to fibroblasts and myofibroblasts, fibrocytes have also been shown to differentiate into other mesenchymal cell types *in vitro *and *in vivo*, including adipocytes, osteoblasts and chondrocytes [23,24].

#### Homing

Fibrocytes use different chemokine ligand - receptor pairs for tissue homing. Human fibrocytes express the chemokine receptors chemokine (C-C motif) receptor (CCR)3, CCR5, CCR7 and chemokine (C-X-C motif) receptor (CXCR)4, whereas mouse fibrocytes express CXCR4, CCR7 and CCR2 [5,6,9,25]. The chemokine receptor - ligand pair CXCR4 - CXCL12 has been shown to play a particularly important role in the homing of fibrocytes [26]. The differential expression of CXCL12 in tissue results in the gradient required for the homing of CXCR4 + cells. Fibrocytes were found to express CXCR4 and to migrate in response to CXCL12 *in vitro *and *in vivo *in a model of bleomycin-induced pulmonary fibrosis [5,27]. In this mouse model, antibody-mediated neutralization of CXCL12 led to reduced fibrocyte recruitment to the lung and decreased collagen deposition [5]. The importance of CXCL12 has also been shown in patients with interstitial lung disease [28]; in this study, not only were the numbers of CD45 + collagen I + CXCR4 + fibrocytes significantly higher than in normal controls, but expression of CXCL12 was markedly elevated in the lung and plasma of patients with lung fibrosis [28]. Other investigators have similarly reported significantly higher levels of CXCL12 in the plasma and lungs of patients with idiopathic pulmonary fibrosis compared with healthy controls [29].

### Fibrotic diseases of the lungs

Fibrotic lung diseases are characterized by varying degrees of inflammation and fibrosis of the lung parenchyma [30], and present clinically as progressive deterioration as the lung tissue is replaced with scar tissue. Idiopathic pulmonary fibrosis is the most common of the fibrotic lung diseases. Aberrant vascular and airway remodeling and fibrosis are also seen in asthma and pulmonary hypertension. Several lines of evidence support the concept of a causal link between the accumulation of fibrocytes at sites of lung tissue injury and the development of lung fibrosis in these diseases.

#### Idiopathic pulmonary fibrosis

The important role that fibrocytes play in the development of lung fibrosis has been reported by several groups using different murine models of lung injury, including those induced by irradiation [31], intrapulmonary bleomycin [5,32] and intrapulmonary fluorescein isothiocyanate (FITC) [25]. In one report, female C57BL/6 mice underwent total lung irradiation, and were subsequently injected with cells from a clonal GFP-positive male bone marrow stromal cell line or male GFP-positive whole bone marrow, then assessed for subsequent lung fibrosis and the contribution of the Y-chromosome probe-positive GFP-positive cells to areas of fibrosis [31]. Labeling of the fibrotic areas revealed that cell division mainly occurred in the cells positive for GFP Y-probe and vimentin, and that these cells were macrophages and fibroblasts, suggesting bone marrow origin [31]. In another study using a bleomycin-induced lung fibrosis model, mice engrafted with bone marrow from transgenic mice expressing GFP had increased numbers of GFP-positive cells in areas of lung fibrosis caused by the bleomycin compared with controls, and flow cytometry of lung cells revealed a significant increase in GFP-positive cells that were also positive for collagen I [32]. Lastly, in another study, investigators treated mice with severe combined immunodeficiency with either bleomycin or saline [5], and subsequently administered human fibrocytes to the mice intravenously. Fibrocytes were shown to preferentially home to the lungs of the mice that were pretreated with bleomycin. In addition, in the immunocompetent mice treated with bleomycin, the magnitude of lung pro-collagen I and III upregulation correlated with the number of fibrocytes in the bone marrow, blood and lung [5]. In this study, levels of CXCL12 were higher in the lungs of the mice treated with bleomycin, and administration of neutralizing anti-CXCL12 antibodies to bleomycin-treated animals led to a reduction in the number of lung fibrocytes and myofibroblasts and reduced lung collagen deposition, while other leukocyte populations in the lung, were not affected [5].

Investigators using the FITC model of lung injury showed that in CCR2-deficient mice challenged with intrapulmonary FITC, fibrocyte recruitment to the lungs was reduced, and wild-type mice that received CCR2^-/- ^bone marrow had similarly reduced fibrocyte recruitment to the lung and lower levels of fibrosis [25]. This protective effect was reversed when bone marrow cells from wild-type mice were transplanted into irradiated CCR2^-/- ^mice. The same investigators also showed that, unlike CCR2^-/- ^mice, CCL2^-/- ^mice are not protected from FITC-induced lung fibrosis, as they are still able to recruit fibrocytes to injured lung tissue, suggesting that CCR2 ligands may play a role in human disease because the recruitment of human fibrocyte precursors (CD14 + CD16- monocytes) into inflamed tissue is dependent on CCR2 [33].

Several groups have reported data regarding the role of fibrocytes in fibrotic interstitial lung disease in humans [28,29]. In one study, investigators compared human lung tissue and peripheral blood from patients with interstitial lung disease with that from normal controls for the presence and number of fibrocytes and their expression of the CXCL12 receptor [28]. Compared with controls, patients with interstitial lung disease had extensive expression of CXCL12 in lung and plasma. Moreover, patients with interstitial lung disease had a number of circulating fibrocytes (collagen-I expressing CD45 + cells) that was an order of magnitude higher than that in normal controls [28]. In a subsequent study, other investigators quantified fibrocytes in the lung tissue, plasma and bronchoalveolar lavage in patients with idiopathic pulmonary fibrosis [29]. They found a positive correlation between the number of lung fibrocytes and the extent of lung fibrosis (*r *= 0.79; *P *< 0.02) and found no fibrocytes in normal lungs. CXCL12 was detectable in the bronchoalveolar fluid in 40% of patients with interstitial lung disease, whereas none was detected in normal controls. Moreover, the concentration of CXCL12 in the plasma of patients with idiopathic pulmonary fibrosis was significantly higher than in healthy controls [29], suggesting that circulating fibrocytes are recruited through the CXCR4 - CXCL12 axis and contribute to lung fibrosis in this patient population. More recently, fibrocytes have been shown to be an indicator of disease activity in patients with idiopathic pulmonary fibrosis [34]. These investigators compared fibrocyte levels in peripheral blood samples in patients with idiopathic pulmonary fibrosis (stable and during an exacerbation), patients with acute respiratory distress syndrome, and normal controls. Fibrocytes were significantly elevated in patients with stable idiopathic pulmonary fibrosis compared with normal controls, and were significantly increased during acute exacerbations of the disease. The number of fibrocytes in patients with acute respiratory distress syndrome was not significantly different from patients with stable idiopathic pulmonary fibrosis or normal controls [34]. Lastly, the mean survival of patients with fibrocyte levels of >5% of total blood leukocytes was 7.5 months compared with 27 months in those with < 5% (*P *< 0.0001).

The concept of fibrocyte CXCR4 regulation as a therapeutic target in pulmonary fibrosis has been recently reported by several groups using the bleomycin-induced model of lung fibrosis [22,35]. One group showed that in this model, fibrocytes homed from the bone marrow to the lungs, and treatment with the mammalian target of rapamycin (mTOR) inhibitor sirolimus attenuated the numbers of fibrocytes in the blood and lungs, and was associated with decreased collagen deposition in the lungs. They also found that CXCR4 was the major chemokine receptor on fibrocytes, and that its expression was induced by hypoxia and via the phosphoinositide 3-kinase (PI3K)/Akt/mTOR pathway [22]. Another group of investigators showed that treatment of mice with AMD3100, a CXCR4 antagonist, resulted in decreased levels of CXCL12 in the bronchoalveolar fluid and decreased numbers of fibrocytes in the lungs [35]. Collagen deposition and pulmonary fibrosis were also attenuated by treatment with AMD3100. Whether pharmacologic inhibition of the CXCR4/CXCL12 axis could modify the lung fibrotic process in humans has yet to be determined, and is an area of active research.

#### Asthma

Recurrent airway inflammation and aberrant remodeling is thought to be the basis of the fibrotic response seen in patients with severe asthma. In one study, fibrocytes were identified in the airways of patients with asthma, and increased in number after antigen challenge [12]. There were increased numbers of CD34 + Col I + cells and few CD34 + α-SMA + cells below the basement membrane in the bronchial mucosa of patients with asthma, and these cells increased significantly within 24 hours after exposure to allergen. In addition, investigators showed that cultures of human CD34 + Col I + cells constitutively expressed α-SMA, and addition of endothelin-1 and TGF-β1 resulted in an increase in fibronectin and collagen III in the culture medium [12]. Others have shown that in bronchial biopsies from patients with mild asthma, cells expressing CD34, CD45 and α-SMA, consistent with fibrocytes, differentiated into myofibroblasts and resulted in increased thickness of the lamina reticularis [36]. Additional evidence that these cells may be of fibrocytic origin comes from a study that showed that CCR7 is expressed in the myofibroblasts in the bronchial mucosa of patients with asthma [37]. Moreover, after observing increased production of CCL19 and its receptor CCR7, these investigators suggested that the CCL19 - CCR7 axis may play a role in the recruitment of fibrocytes to the lungs in patients with asthma [37]. Fibrocytes were also shown to be increased in patients with asthma who have chronic airway obstruction; in this study, the percentage of bone marrow-derived fibrocytes in the peripheral blood was higher in patients with chronic obstructive asthma than in patients with asthma without chronic airway obstruction or normal controls [38]. The investigators also showed that the yearly decline in lung function was significantly associated with the percentage of circulating fibrocytes in patients with chronic obstructive asthma. Recently, investigators have reported that in patients with severe refractory asthma, the number of fibrocytes was significantly increased in the lamina propria compared with normal controls, and was also increased in the peripheral blood [39].

#### Pulmonary hypertension

Pulmonary vascular remodeling is associated with fibroproliferative changes in the media and adventitia, and with increased extracellular matrix deposition. Although resident fibroblasts have been considered the primary contributor to this process, more recently, the importance of bone marrow-derived fibrocytes in this process has become apparent. In one study, the bone marrow of GFP transgenic mice was transplanted into lethally irradiated syngeneic mice [40]. After 8 weeks, the chimera mice were exposed to chronic hypoxia. Compared with controls, marked vascular remodeling including medial hypertrophy and adventitial thickening was observed in the pulmonary arteries of the mice, and an abundance of GFP-positive cells were observed in the pulmonary arterial wall of the mice with hypoxia-induced pulmonary hypertension. Most of these GFP-positive cells also expressed α-SMA, indicating a myofibroblast phenotype. The investigators concluded that bone-marrow-derived cells mobilized to the hypertensive pulmonary arteries and contributed to the pulmonary vascular remodeling. Others have also shown that circulating mesenchymal progenitor cells contribute to hypoxia-induced pulmonary vascular remodeling in animal models [41]. The contribution of circulating fibrocytes was confirmed using depletion studies, and showed that a reduction in fibrocytes in the circulation of chronically hypoxic rats resulted in a marked attenuation of adventitial thickening and fibrosis.

### Fibrotic diseases of the heart

Fibrosis has been shown to be an important factor associated with the adverse ventricular remodeling seen in ischemic cardiomyopathy [42-44], intimal hyperplasia after revascularization [45], and atherosclerosis [46,47]. Fibrosis also appears to be important in the pathophysiology of myxomatous valve disease [48,49] and transplant vasculopathy [50].

#### Myxomatous valve disease

The data supporting the role of fibrocytes in myxomatous valve disease come mainly from two studies. In the first study, investigators used immunohistochemical analysis to compare 14 mitral valves removed for myxomatous degeneration with 11 normal mitral valves removed at autopsy, and found that myxomatous valves contained both vimentin and α-SMA, and had features of activated myofibroblasts [49]. In the second study, investigators compared the stromal cells within myxomatous mitral valves and those within normal valves, and found that the stromal cells in myxomatous valves were not only increased in number, but also appeared hyperplastic, were CD34 +, and had multipolar cytoplasmic processes consistent with fibrocytes [48].

#### Intimal hyperplasia

Intimal hyperplasia, a common response of blood vessels to mechanical injury, results in the thickening of the tunica intima. It has been shown to be an important cause of saphenous vein graft disease after bypass surgery, and of recurrent stenoses after percutaneous revascularization of coronary and peripheral arteries. In an *in vivo *ovine model of carotid artery synthetic patch graft, circulating leukocytes were shown to produce collagen and α-SMA, and these cells were subsequently found by immunohistochemistry to express a set of markers unique to fibrocytes, namely, CD34, CD45, vimentin and α-SMA [45]. These same investigators took monocytes from human volunteers and cultured them; the cultured cells expressed markers consistent with fibrocytes (CD34 + CD45 + Col I + α-SMA +) and were morphometrically similar to the vascular smooth muscle cells found in intimal hyperplasia [45], suggesting an association between intimal hyperplasia and fibrocyte migration.

#### Atherosclerosis

In a rabbit model of atherosclerosis, CD34 + progenitor cells were identified within and overlying atherosclerotic plaques in rabbits fed a high cholesterol diet compared with controls. These cells stained positive for CD45, RAM-11, prolyl-4 hydroxylase and α-SMA, suggesting a hematopoietic origin and a fibroblast/myofibroblast phenotype [47]. Other investigators evaluating human carotid endarterectomy specimens found evidence of fibrocytes on immunohistochemistry staining, and suggested that fibrocytes contribute to the formation and stability of the fibrous cap [46]. Specifically, fibrocytes were found in areas of plaque growth and healing. The authors further characterized them to be spindle-shaped, collagen-producing and colocalizing with TGF-β (which, as noted before, is known to promote fibrocyte formation) [46].

#### Ischemic cardiomyopathy

Deposition and remodeling of connective tissue plays an important role in cardiac repair after injury, and it is generally considered that both reactive and reparative fibrosis contributes to the adverse remodeling seen in ischemic cardiomyopathy [44]. Several investigators have evaluated the potential role of fibrocytes in this process. In a closed chest mouse model of ischemia - reperfusion cardiomyopathy, increased numbers of small spindle-shaped cells were noted in the myocardium, and these cells expressed collagen I, α-SMA, CD34 and CD45 [44]. Moreover, after administration of serum amyloid P, the number of these myocardial spindle-shaped cells was decreased, and the ischemia - reperfusion-induced fibrosis and subsequent ventricular dysfunction were prevented [44]. In a separate study, investigators determined whether bone-marrow-derived cells contributed to cardiac fibrosis in the chronically failing heart, and whether certain chemokines played a role [43]. In another study, investigators used the *mammalian sterile 20-like kinase *(*Mst*) *1 *transgenic mouse, in which overexpression of *Mst1 *results in caspase activation, increased apoptosis and dilated cardiomyopathy. In this model, bone-marrow-derived cells were recruited in significantly greater numbers in *Mst1 *versus control mice. In addition, the investigators found that myocytes constitutively secreted CXCL12, which was shown to increase cardiac fibroblast migration by 59%, suggesting that the recruitment of bone-marrow-derived cells plays an important role in the pathogenesis of cardiac fibrosis in heart failure, and that CXCL12 is involved in this recruitment [43]. Lastly, while studying the role of CXCL10 in cardiac repair after myocardial infarction, investigators found that both CXCL10-null and wild-type mice had comparable infarct size, but the CXCL10-null mice exhibited a hypercellular early reparative response, delayed contraction of scar, early dilation, and wall thinning [42]. Interestingly, the absence of CXCL10 was associated with intense infiltration with CD45 + α-SMA + cells and with reduced recruitment of cells that expressed CXCR3, the receptor for CXCL10. These findings suggest that although CXCL10 may be important in infarct healing, wound contraction and favorable remodeling, its effects may be primarily due its direct effects on fibroblast migration and function [42].

## Conclusion

The fibrocyte is a unique mesenchymal progenitor cell found to be an important source of fibroblasts and myofibroblasts during tissue repair and remodeling. In some disease states, elevated circulating levels of fibrocytes are associated with increased fibrosis and adverse clinical outcomes. Gaining a better understanding of this important cell has the potential to allow development of therapeutic strategies to attenuate its negative effects in fibrotic diseases.

## Competing interests

The authors declare that they have no competing interests.

## Authors' contributions

ECK wrote the first draft of the manuscript. BM and RMS provided constructive criticism, and contributed substantially to the second and final draft of the manuscript. All authors read and approved the final manuscript.

## References

[B1] Bucala R Spiegel LA Chesney J Hogan M Cerami A Circulating fibrocytes define a new leukocyte subpopulation that mediates tissue repair Mol Med 1994 1 71 81 8790603 PMC2229929

[B2] Herzog EL Bucala R Fibrocytes in health and disease Exp Hematol 2010 38 548 556 10.1016/j.exphem.2010.03.004 20303382 PMC3136351

[B3] Kizer JR Zisman DA Blumenthal NP Kotloff RM Kimmel SE Strieter RM Arcasoy SM Ferrari VA Hansen-Flaschen J Association between pulmonary fibrosis and coronary artery disease Arch Intern Med 2004 164 551 556 10.1001/archinte.164.5.551 15006833

[B4] Metz CN Fibrocytes: a unique cell population implicated in wound healing Cell Mol Life Sci 2003 60 1342 1350 10.1007/s00018-003-2328-0 12943223 PMC11138753

[B5] Phillips RJ Burdick MD Hong K Lutz MA Murray LA Xue YY Belperio JA Keane MP Strieter RM Circulating fibrocytes traffic to the lungs in response to CXCL12 and mediate fibrosis J Clin Invest 2004 114 438 446 15286810 10.1172/JCI20997 PMC484979

[B6] Quan TE Cowper S Wu SP Bockenstedt LK Bucala R Circulating fibrocytes: collagen-secreting cells of the peripheral blood Int J Biochem Cell Biol 2004 36 598 606 10.1016/j.biocel.2003.10.005 15010326

[B7] Chesney J Metz C Stavitsky AB Bacher M Bucala R Regulated production of type I collagen and inflammatory cytokines by peripheral blood fibrocytes J Immunol 1998 160 419 425 9551999

[B8] Chesney J Bacher M Bender A Bucala R The peripheral blood fibrocyte is a potent antigen-presenting cell capable of priming naive T cells in situ Proc Natl Acad Sci USA 1997 94 6307 6312 10.1073/pnas.94.12.6307 9177213 PMC21045

[B9] Abe R Donnelly SC Peng T Bucala R Metz CN Peripheral blood fibrocytes: differentiation pathway and migration to wound sites J Immunol 2001 166 7556 7562 11390511 10.4049/jimmunol.166.12.7556

[B10] Hartlapp I Abe R Saeed RW Peng T Voelter W Bucala R Metz CN Fibrocytes induce an angiogenic phenotype in cultured endothelial cells and promote angiogenesis *in vivo* FASEB J 2001 15 2215 2224 10.1096/fj.01-0049com 11641248

[B11] Pilling D Fan T Huang D Kaul B Gomer RH Identification of markers that distinguish monocyte-derived fibrocytes from monocytes, macrophages, and fibroblasts PLoS One 2009 4 e7475 10.1371/journal.pone.0007475 19834619 PMC2759556

[B12] Schmidt M Sun G Stacey MA Mori L Mattoli S Identification of circulating fibrocytes as precursors of bronchial myofibroblasts in asthma J Immunol 2003 171 380 389 12817021 10.4049/jimmunol.171.1.380

[B13] Pilling D Buckley CD Salmon M Gomer RH Inhibition of fibrocyte differentiation by serum amyloid P J Immunol 2003 171 5537 5546 14607961 10.4049/jimmunol.171.10.5537 PMC4482350

[B14] Pilling D Roife D Wang M Ronkainen SD Crawford JR Travis EL Gomer RH Reduction of bleomycin-induced pulmonary fibrosis by serum amyloid P J Immunol 2007 179 4035 4044 17785842 10.4049/jimmunol.179.6.4035 PMC4482349

[B15] Hong KM Belperio JA Keane MP Burdick MD Strieter RM Differentiation of human circulating fibrocytes as mediated by transforming growth factor-beta and peroxisome proliferator-activated receptor gamma J Biol Chem 2007 282 22910 22920 10.1074/jbc.M703597200 17556364

[B16] Shao DD Suresh R Vakil V Gomer RH Pilling D Pivotal Advance: Th-1 cytokines inhibit, and Th-2 cytokines promote fibrocyte differentiation J Leukoc Biol 2008 83 1323 1333 10.1189/jlb.1107782 18332234 PMC2659591

[B17] Wang J Jiao H Stewart TL Shankowsky HA Scott PG Tredget EE Improvement in postburn hypertrophic scar after treatment with IFN-alpha2b is associated with decreased fibrocytes J Interferon Cytokine Res 2007 27 921 930 10.1089/jir.2007.0008 18052725

[B18] Vannella KM McMillan TR Charbeneau RP Wilke CA Thomas PE Toews GB Peters-Golden M Moore BB Cysteinyl leukotrienes are autocrine and paracrine regulators of fibrocyte function J Immunol 2007 179 7883 7890 18025235 10.4049/jimmunol.179.11.7883 PMC3646370

[B19] Pilling D Tucker NM Gomer RH Aggregated IgG inhibits the differentiation of human fibrocytes J Leukoc Biol 2006 79 1242 1251 10.1189/jlb.0805456 16543402 PMC4482138

[B20] Vakil V Sung JJ Piecychna M Crawford JR Kuo P Abu-Alfa AK Cowper SE Bucala R Gomer RH Gadolinium-containing magnetic resonance image contrast agent promotes fibrocyte differentiation J Magn Reson Imaging 2009 30 1284 1288 10.1002/jmri.21800 19937928 PMC2787835

[B21] Mori L Bellini A Stacey MA Schmidt M Mattoli S Fibrocytes contribute to the myofibroblast population in wounded skin and originate from the bone marrow Exp Cell Res 2005 304 81 90 10.1016/j.yexcr.2004.11.011 15707576

[B22] Mehrad B Burdick MD Strieter RM Fibrocyte CXCR4 regulation as a therapeutic target in pulmonary fibrosis Int J Biochem Cell Biol 2009 41 1708 1718 10.1016/j.biocel.2009.02.020 19433312 PMC2681415

[B23] Hong KM Burdick MD Phillips RJ Heber D Strieter RM Characterization of human fibrocytes as circulating adipocyte progenitors and the formation of human adipose tissue in SCID mice FASEB J 2005 19 2029 2031 16188961 10.1096/fj.05-4295fje

[B24] Choi YH Burdick MD Strieter RM Human circulating fibrocytes have the capacity to differentiate osteoblasts and chondrocytes Int J Biochem Cell Biol 2010 42 662 671 10.1016/j.biocel.2009.12.011 20034590 PMC2835809

[B25] Moore BB Kolodsick JE Thannickal VJ Cooke K Moore TA Hogaboam C Wilke CA Toews GB CCR2-mediated recruitment of fibrocytes to the alveolar space after fibrotic injury Am J Pathol 2005 166 675 684 15743780 10.1016/S0002-9440(10)62289-4 PMC1780139

[B26] Murdoch C CXCR4: chemokine receptor extraordinaire Immunol Rev 2000 177 175 184 10.1034/j.1600-065X.2000.17715.x 11138774

[B27] Strieter RM Gomperts BN Keane MP The role of CXC chemokines in pulmonary fibrosis J Clin Invest 2007 117 549 556 10.1172/JCI30562 17332882 PMC1804376

[B28] Mehrad B Burdick MD Zisman DA Keane MP Belperio JA Strieter RM Circulating peripheral blood fibrocytes in human fibrotic interstitial lung disease Biochem Biophys Res Commun 2007 353 104 108 10.1016/j.bbrc.2006.11.149 17174272

[B29] Andersson-Sjoland A de Alba CG Nihlberg K Becerril C Ramirez R Pardo A Westergren-Thorsson G Selman M Fibrocytes are a potential source of lung fibroblasts in idiopathic pulmonary fibrosis Int J Biochem Cell Biol 2008 40 2129 2140 10.1016/j.biocel.2008.02.012 18374622

[B30] American Thoracic Society/European Respiratory Society International Multidisciplinary Consensus Classification of the Idiopathic Interstitial Pneumonias Am J Respir Crit Care Med 2002 165 277 304 11790668 10.1164/ajrccm.165.2.ats01

[B31] Epperly MW Guo H Gretton JE Greenberger JS Bone marrow origin of myofibroblasts in irradiation pulmonary fibrosis Am J Respir Cell Mol Biol 2003 29 213 224 10.1165/rcmb.2002-0069OC 12649121

[B32] Hashimoto N Jin H Liu T Chensue SW Phan SH Bone marrow-derived progenitor cells in pulmonary fibrosis J Clin Invest 2004 113 243 252 14722616 10.1172/JCI18847 PMC310750

[B33] Moore BB Murray L Das A Wilke CA Herrygers AB Toews GB The role of CCL12 in the recruitment of fibrocytes and lung fibrosis Am J Respir Cell Mol Biol 2006 35 175 181 10.1165/rcmb.2005-0239OC 16543609 PMC2643255

[B34] Moeller A Gilpin SE Ask K Cox G Cook D Gauldie J Margetts PJ Farkas L Dobranowski J Boylan C O'Byrne PM Strieter RM Kolb M Circulating fibrocytes are an indicator of poor prognosis in idiopathic pulmonary fibrosis Am J Respir Crit Care Med 2009 179 588 594 10.1164/rccm.200810-1534OC 19151190

[B35] Song JS Kang CM Kang HH Yoon HK Kim YK Kim KH Moon HS Park SH Inhibitory effect of CXC chemokine receptor 4 antagonist AMD3100 on bleomycin induced murine pulmonary fibrosis Exp Mol Med 2010 42 465 472 10.3858/emm.2010.42.6.048 20498529 PMC2892600

[B36] Nihlberg K Larsen K Hultgardh-Nilsson A Malmstrom A Bjermer L Westergren-Thorsson G Tissue fibrocytes in patients with mild asthma: a possible link to thickness of reticular basement membrane? Respir Res 2006 7 50 10.1186/1465-9921-7-50 16571120 PMC1458331

[B37] Kaur D Saunders R Berger P Siddiqui S Woodman L Wardlaw A Bradding P Brightling CE Airway smooth muscle and mast cell-derived CC chemokine ligand 19 mediate airway smooth muscle migration in asthma Am J Respir Crit Care Med 2006 174 1179 1188 10.1164/rccm.200603-394OC 16959919

[B38] Wang CH Huang CD Lin HC Lee KY Lin SM Liu CY Huang KH Ko YS Chung KF Kuo HP Increased circulating fibrocytes in asthma with chronic airflow obstruction Am J Respir Crit Care Med 2008 178 583 591 10.1164/rccm.200710-1557OC 18583572

[B39] Saunders R Siddiqui S Kaur D Doe C Sutcliffe A Hollins F Bradding P Wardlaw A Brightling CE Fibrocyte localization to the airway smooth muscle is a feature of asthma J Allergy Clin Immunol 2009 123 376 384 10.1016/j.jaci.2008.10.048 19081612 PMC3992369

[B40] Hayashida K Fujita J Miyake Y Kawada H Ando K Ogawa S Fukuda K Bone marrow-derived cells contribute to pulmonary vascular remodeling in hypoxia-induced pulmonary hypertension Chest 2005 127 1793 1798 10.1378/chest.127.5.1793 15888860

[B41] Frid MG Brunetti JA Burke DL Carpenter TC Davie NJ Reeves JT Roedersheimer MT van Rooijen N Stenmark KR Hypoxia-induced pulmonary vascular remodeling requires recruitment of circulating mesenchymal precursors of a monocyte/macrophage lineage Am J Pathol 2006 168 659 669 10.2353/ajpath.2006.050599 16436679 PMC1606508

[B42] Bujak M Dobaczewski M Gonzalez-Quesada C Xia Y Leucker T Zymek P Veeranna V Tager AM Luster AD Frangogiannis NG Induction of the CXC chemokine interferon-gamma-inducible protein 10 regulates the reparative response following myocardial infarction Circ Res 2009 105 973 983 10.1161/CIRCRESAHA.109.199471 19797174 PMC2783369

[B43] Chu PY Mariani J Finch S McMullen JR Sadoshima J Marshall T Kaye DM Bone marrow-derived cells contribute to fibrosis in the chronically failing heart Am J Pathol 2010 176 1735 1742 10.2353/ajpath.2010.090574 20150435 PMC2843465

[B44] Haudek SB Xia Y Huebener P Lee JM Carlson S Crawford JR Pilling D Gomer RH Trial J Frangogiannis NG Entman ML Bone marrow-derived fibroblast precursors mediate ischemic cardiomyopathy in mice Proc Natl Acad Sci USA 2006 103 18284 18289 10.1073/pnas.0608799103 17114286 PMC1643845

[B45] Varcoe RL Mikhail M Guiffre AK Pennings G Vicaretti M Hawthorne WJ Fletcher JP Medbury HJ The role of the fibrocyte in intimal hyperplasia J Thromb Haemost 2006 4 1125 1133 10.1111/j.1538-7836.2006.01924.x 16689767

[B46] Medbury HJ Tarran SL Guiffre AK Williams MM Lam TH Vicaretti M Fletcher JP Monocytes contribute to the atherosclerotic cap by transformation into fibrocytes Int Angiol 2008 27 114 123 18427397

[B47] Zulli A Buxton BF Black MJ Hare DL CD34 Class III positive cells are present in atherosclerotic plaques of the rabbit model of atherosclerosis Histochem Cell Biol 2005 124 517 522 10.1007/s00418-005-0072-2 16177890

[B48] Barth PJ Koster H Moosdorf R CD34 + fibrocytes in normal mitral valves and myxomatous mitral valve degeneration Pathol Res Pract 2005 201 301 304 10.1016/j.prp.2005.02.001 15991836

[B49] Rabkin E Aikawa M Stone JR Fukumoto Y Libby P Schoen FJ Activated interstitial myofibroblasts express catabolic enzymes and mediate matrix remodeling in myxomatous heart valves Circulation 2001 104 2525 2532 10.1161/hc4601.099489 11714645

[B50] Onuta G van Ark J Rienstra H Boer MW Klatter FA Bruggeman CA Zeebregts CJ Rozing J Hillebrands JL Development of transplant vasculopathy in aortic allografts correlates with neointimal smooth muscle cell proliferative capacity and fibrocyte frequency Atherosclerosis 2010 209 393 402 10.1016/j.atherosclerosis.2009.10.020 19913790

